# EDTA salt modified carbon paste electrode for square wave voltammetric determination of theophylline in pharmaceutical tablet formulation

**DOI:** 10.1371/journal.pone.0255700

**Published:** 2022-06-10

**Authors:** Amsalu Moges, Mulugeta Dawit, Mahilet Turbale, Meareg Amare

**Affiliations:** 1 Debre Markos University, Debre Markos, Ethiopia; 2 Debre Tabor University, Debra Tabor, Ethiopia; 3 Samara University, Samara, Ethiopia; 4 Bahir Dar University, Bahir Dar, Ethiopia; Bhagwan Mahvir College of Pharmacy, INDIA

## Abstract

In this study, a square wave voltammetric method for determination of theophylline in tablet formulation based on EDTA salt modified carbon paste electrode is presented. CV, FT-IR, and EIS results confirmed modification of the carbon paste with EDTA salt. In contrast to the unmodified carbon paste electrode, the modified carbon paste electrode showed irreversible oxidation of theophylline with considerable current enhancement. Investigation of the effect of scan rate on the Ip and Ep response of the modified electrode for theophylline revealed predominantly diffusion controlled oxidation kinetics. Under the optimized conditions, square wave oxidative peak current of theophylline in pH 7.0 PBS showed linear dependence on concentration in the range 10–200 μM with determination coefficient (R^2^), limit of detection, and limit of quantification of 0.99782, 0.0257 μM, and 0.0857 μM, respectively. Detection of an amount of theophylline in the analyzed tablet formulation with 1.85% error from its nominal content (120 mg/tablet) confirmed the accuracy of the developed method. Spike and interference recovery results of 98.59%, and 95.7–100%, respectively validated the applicability of the developed method for determination of theophylline content in tablet samples.

## 1. Introduction

**Fig 1:**
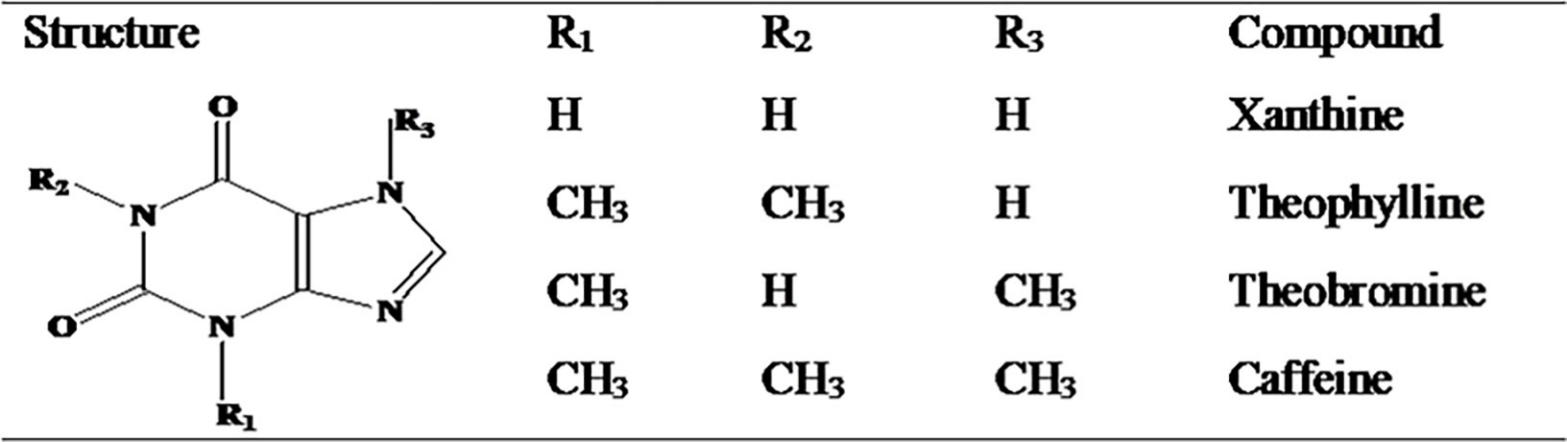
Structure of xanthine and its naturally occurring N-methyl derivatives.

The N-methyl derivatives of xanthine, including theophylline (1, 3-dimethyl-3,7-dihydro-1H-purin-2,6-dion), theobromine (3,7-dihydro-3,7-dimethyl- 1H-purine-2,6-dione), and caffeine (3,7-dihydro-1,3,7-trimethyl- 1H-purine-2,6-dione) (Fig 1), are alkaloids widely distributed in plant products and beverages such as tea, coffee, and cocoa beans. These are known to exhibit physiological effects such as gastric acid secretion and stimulation of the central nervous system [1].

Theophylline (TP), which is effective in the treatment of respiratory diseases is prescribed in the therapy of asthma and chronic obstructive pulmonary disorder in adults [2]. Moreover, TP is mainly used to treat emphysema, bronchial asthma, cardiac difficulty breathing, bronchitis, chronic obstructive pulmonary disease (COPD), apnea, and bradycardia [3]. The accepted plasma TP level in adults being 5–20 μg/mL, while dosage lower than the accepted level is non-therapeutic [4], its higher levels or excessive administration occasionally causes serious toxicity leading to vomiting, tachycardia, seizures, central nervous system excitation, increased heart rate, diarrhea, anxiety, restlessness, and dizziness [1, 3, 5]. Although are generally not apparent to patients, TP has measurable neuropsychological and metabolic effects similar to those associated with caffeine [6]. These all together escort monitoring the level of TP in pharmaceutical formulations and human serum samples using a sensitive and selective method.

UV-Vis spectrophotometry [7], high-performance liquid chromatography [8], liquid chromatography-mass spectrometry [9], and surface-enhanced Raman scattering (SERS) sensor [10–12] are among the common techniques reported for determination of TP. However, almost all these techniques are known to have several drawbacks including low sensitivity, long analysis time, tedious sample preparation, expensive instruments and maintenance, trained technician, some requiring derivatization procedure before determination, and use of organic solvents inducing environmental pollution [13–15]. Compared to the reported conventional methods, electrochemical methods require relatively minimum cost, high sensitivity and swift technique for bio-molecule detection [14, 16–19].

Attempts have been made on determination of TP using glassy carbon electrode [20–23], and carbon paste electrode [24] modified with various materials. Among the carbon based electrodes, carbon paste electrode is the most available, easily prepared, and hence widely reported for its electrochemical sensor application [25, 26]. Modification of carbon paste electrode commonly improves the sensitivity, selectivity, and reproducibility of the method [27–29]. Most of the reported modifiers being expensive, less available, and or requiring complex modification steps [1, 30], development of a voltammetric method based on carbon paste electrode modified with relatively cheap, available, easy modification steps, and selective material is crucial. To the best of our knowledge, ethylenediaminetetraacetic acid salt (EDTA salt) modified carbon paste electrode has not been reported for determination of theophylline.

Thus, the present work demonstrates an accurate, selective, and sensitive square wave voltammetric method based on EDTA salt modified carbon past electrode for determination of theophylline in tablet sample.

## 2. Materials and methods

### 2.1. Chemicals and reagents

EDTA salt (99%, Abron chemicals), theophylline anhydrous (99.70%, Addis pharmaceutical factory, Ethiopia), graphite powder (>99.5%, Blulux laboratories Pvt. Ltd), HNO_3_ (70%, Nice Chemicals Pvt. Ltd, India), NaH_2_PO_4_ & Na_2_HPO_4_ (both 98–101%, Sisco Research Laboratories Pvt. Ltd), NaOH (99%, lab tech chemicals), HCl (36%, Loba Chemie, India), paraffin oil (BDH laboratory supplies, England), K_3_Fe(CN)_6 &_ K_4_Fe(CN)_6_.3H_2_O) (both ≥99.0%, Sigma Aldrich), KCl (99.5%, Aldrich), ascorbic acid (99%, Blulux Laboratories reagent Pvt. Ltd, India), uric acid and caffeine anhydrous (both 99%, Loba Chemie, India) were used. Distilled water was used throughout the experiments.

### 2.2. Apparatus and instruments

CHI760E Electrochemical Workstation (Austin, Texas, USA), pH meter (Adwa, AD 8000, pH/mv/EC/TDS), electronic balance (Nimbus), and FT-IR (PerkinElmer FT-IR spectrometer, USA) were among the instruments/apparatus used in the present work.

### 2.3. Procedures

#### 2.3.1. Electrochemical measurements

A conventional three-electrode system comprising unmodified carbon paste electrode (UCPE) or EDTA salt modified carbon paste electrode (MCPE) as working electrode, Ag/AgCl (3.0 M) as reference electrode, and platinum coil as auxiliary electrode was employed.

#### 2.3.2. Preparation of working electrodes

UCPE was prepared following a procedure reported elsewhere [31]. Briefly: A mixture of 7.0 g of graphite powder and 3.0 g of paraffin oil (70:30 w/w%), homogenized thoroughly with a mortar and pestle for 40 minutes, was allowed to rest for 24 h. The paste was then packed into a plastic syringe at the back of which copper wire was introduced to provide electrical contact and finally smoothed on a white paper before use. Carbon past modified with various amounts of EDTA salt were also prepared with slight modification of reported procedure [23]. Briefly: A mixture of 7.0 g of graphite powder and 0.5 g EDTA salt (5%) put on a small agate mortar was agitated for about 5 minutes. To the mixture, 3.0 g of paraffin oil was added and milled for additional 40 minutes. The homogenized paste was then allowed to rest for 24 h. Furthermore, the same procedure was followed to prepare three other carbon paste electrodes modified with EDTA salt of 1.0 g, 2.5 g, and 4.0 g with the objective to study the EDTA salt loading on the current response for TP [32].

#### 2.3.3. Preparation of standard theophylline solutions

A 25 mM stock solution of TP in 100 mL flask was prepared by dissolving an accurately weighed 0.4505 g of anhydrous theophylline in distilled water and stored under refrigeration [30]. An intermediate working standard solution of 1.0 mM in pH 7.0 PBS was prepared from the stock solution. Calibration TP standard solutions (10, 20, 40, 60, 80, 100, 150, and 200 μM) in pH 7.0 PBS were also prepared from the intermediate solution through serial dilution.

#### 2.3.4. Tablet sample preparation

Six randomly selected theophylline tablets (Addis pharmaceutical factory (APF) brand), declared 120 mg/tablet with an average mass of 300 mg per tablet, were powdered using mortar and pestle. A portion of accurately 18 mg powder was transferred into a 100 mL volumetric flask, and filled up to the mark with distilled water [30]. Tablet sample for analysis was then prepared by transferring 5 mL of the tablet aliquot in to 50 mL volumetric flask and filled up to the mark with pH 7.0 PBS. Eight tablet sample solutions were also prepared following the same procedure for recovery and interference studies. Of the eight tablet solutions, while one was left unspiked that served as a reference for both spike and interference studies, the second was spiked with 40 μM for recovery test, and the remaining six tablet solutions spiked with either 20 or 40 μM of ascorbic acid (AA), caffeine (Caf), or uric acid (UA) were used for interference study.

## 3. Results and discussion

### 3.1. Characterization of the modified electrode

Electrochemical impedance spectroscopy (EIS), cyclic voltammetry (CV), and Fourier transform infrared (FT-IR) spectroscopic techniques were employed to characterize the EDTA salt modified CPE.

#### 3.1.1. CV and EIS characterization

**Fig 2:**
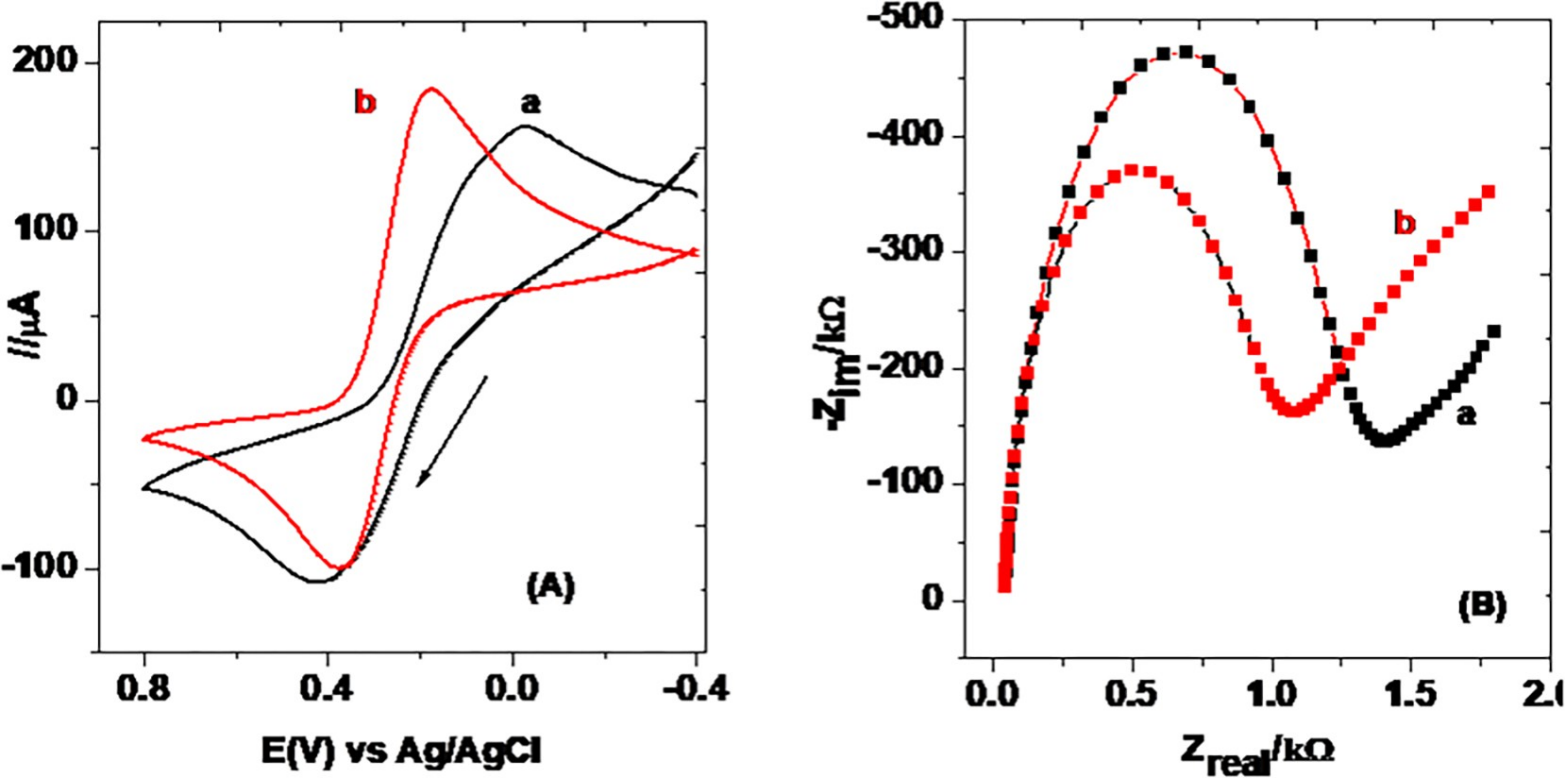
(A) cyclic voltammograms of 10.0 mM [Fe (CN)_6_]^3-/4-^ mixture in pH 7.0 PBS containing 0.1 M KCl at (a) UCPE, and (b) MCPE, and (B) Nyquist plots of UCPE (a) and MCPE (b) in 10.0 mM [Fe (CN)_6_]^3-/4-^ mixture in pH 7.0 PBS containing 0.1 M KCl at frequency range: 0.01–100,000 Hz, applied potential: +0.23 V, and amplitude: 0.01 V.

A mixture of 10 mM [Fe (CN)_6_]^3-/4-^ in pH 7.0 PBS containing 0.1 M KCl was used as a probe for the characterization of the EDTA salt/CPE (MCPE) using both the EIS and CV techniques. Fig 2 presents the cyclic voltammograms (A) and Nyquist plots (B) of the UCPE (curve a) and MCPE (curve b).

Appearance of peaks in opposite scan directions with comparable current at both the UCPE and MCPE is typical of the probe (Fig 2A). In contrast to the peak-peak separation of 410 mV for the probe observed at the UCPE (curve a), appearance of oxidative and reductive peaks with much reduced peak-peak separation of 191 mV at the MCPE (curve b) evidenced modification of the electrode. The observed over-potential reduction of the probe at the MCPE might be ascribed to possibly improved conductivity of the modifier.

The Nyquist plots (Fig 2B) for both UCPE (curve a) and MCPE (curve b) comprised of two sections; a linear line about 45^0^ at low frequency region indicating the diffusion mass transfer of the electroactive species and a semi-circle of different diameter in the higher frequency region representing the surface electron exchange environment. In contrast to the UCPE, a semi-circle with a smaller diameter at the MCPE signified an improved charge transfer at the surface of the modified electrode which is in good agreement with the CV result [33]. The values of selected circuit elements (solution resistance (R_s_), charge transfer resistance (R_ct_), and double layer capacitance (C_dl_)) calculated for both UCPE and MCPE using Eq (1) [34] are shown in Table 1.


$$C_{dl}=\frac{1}{2\pi R_{ct}}$$ (1)

**Table 1:** Summary of calculated equivalent circuit parameters for the UCPE and MCPE.

Electrodes	R_s_ (Ω)	R_ct_ (Ω)	C_dl_ (μF)
UCPE	46	1432	5.29 ×10^−8^
MCPE	46	1080	6.23 × 10^−8^

The appearance of the redox peaks of the probe (Fig 2) with reduced over potential at the MCPE (curve a of Inset) than at the UCPE (curve a of Inset) may thus be attributed to the lower charge transfer resistance (R_ct_) value and hence improved conductivity of the MCPE.

#### 3.1.2. FT-IR spectroscopic characterization

**Fig 3:**
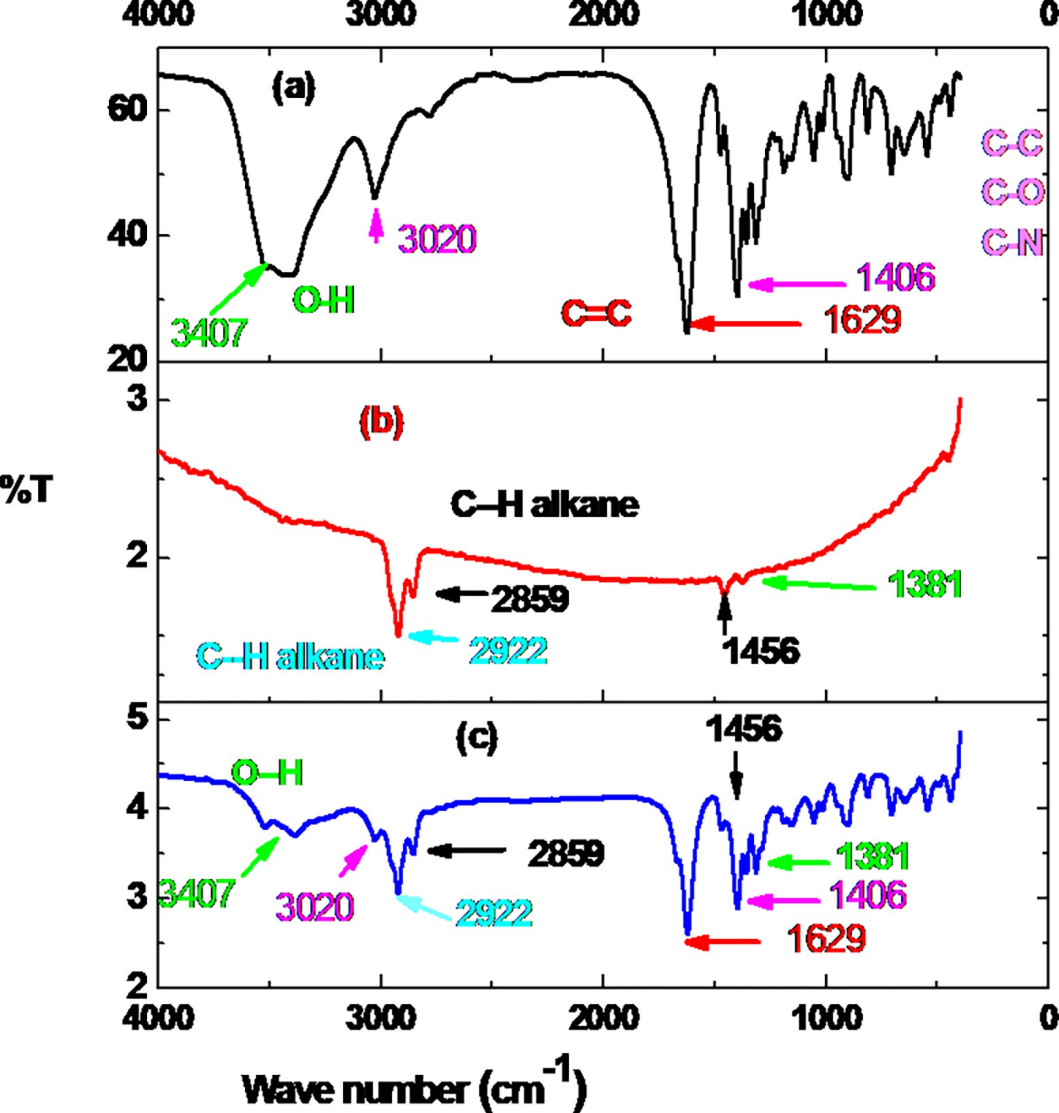
FT-IR spectra of (a) EDTA salt, (b) CPE, and (c) MCPE.

To further confirm the modification of carbon paste with EDTA salt, FT-IR spectra of EDTA salt, UCPE, and MCPE were recoded (Fig 3). From the EDTA salt spectrum (curve a), vibrational bands around 3407 cm^-1^ assigned for O-H stretching, around 3020 cm^-1^ assigned for unidentified functional group, at 1629 cm^-1^ assigned for C = C, and 1406 cm^-1^ assigned for C-H bending, C-C, and C-N [35] are all retained in the MCPE spectrum (curve c) although with varying intensities. Moreover, the vibrational bands between 2922–3000 cm^-1^ which are characteristic for alkane C-H stretching, at 1456 cm^-1^ assigned for alkane C-H bending [35] at the CPE (curve b) also appeared at MCPE spectrum still with varying intensity. Although with differing intensity and frequency, appearance of all characteristic vibrational bands of separated EDTA salt (curve a) and CPE (curve b) in the spectrum for the mixture (curve c) thus confirmed successful modification of the CPE with the EDTA salt.

### 3.2. Cyclic voltammetric investigation of TP at MCPE

Cyclic voltammetry was employed to investigate the electrochemical behavior of TP, effect of scan rate and solution pH on both the peak current and peak potential of TP, all at MCPE.

#### 3.2.1. Cyclic voltammetric behavior of TP at MCPE

**Fig 4:**
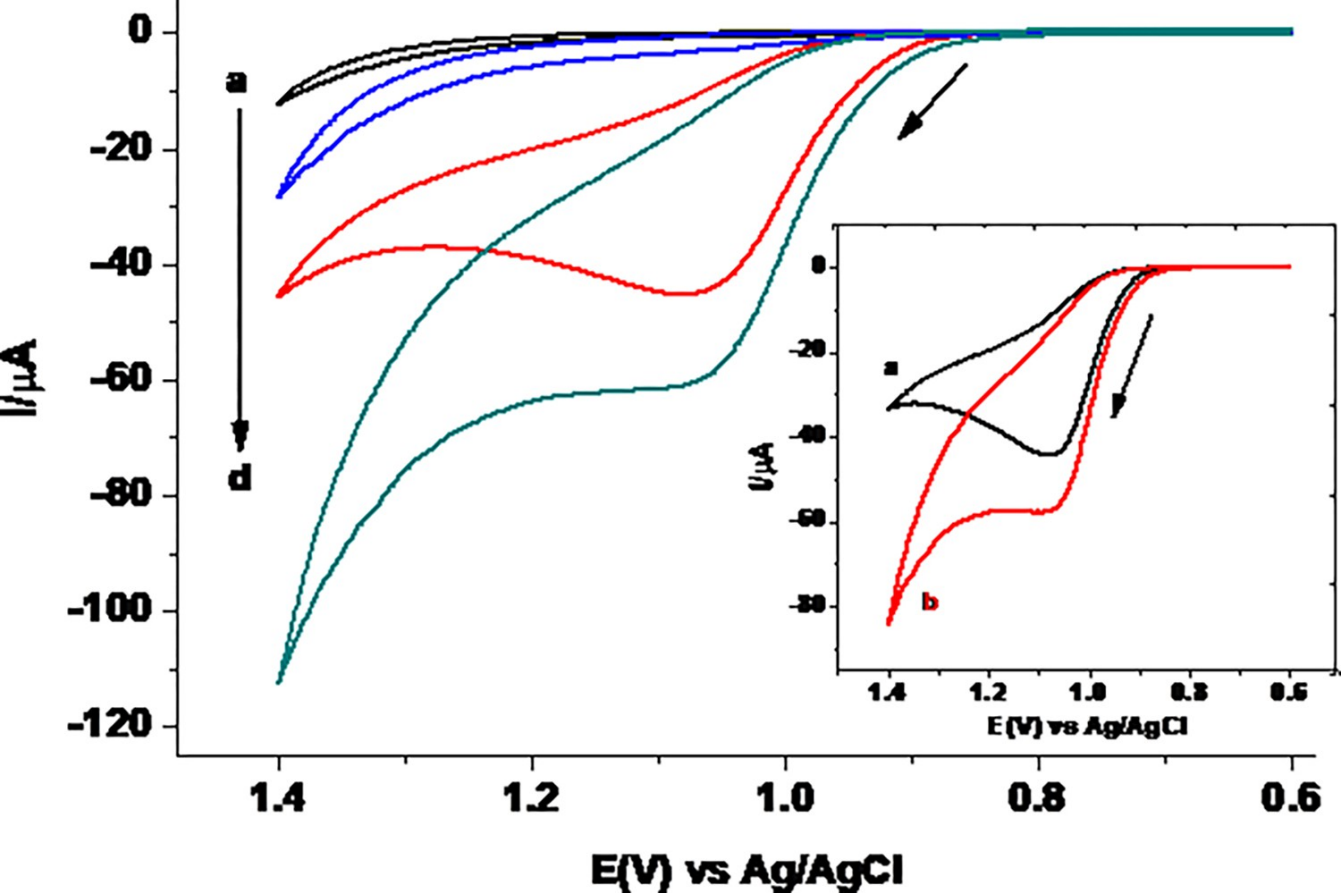
Cyclic voltammograms of UCPE (a & c) and MCPE (b & d) in pH 7.0 PBS in the absence (a & b) and presence (c and d) of 0.5 mM TP at scan rate of 100 mV s^-1^. Inset: Corrected for blank cyclic voltammograms of (a) UCPE and (b) MCPE.

Fig 4 presents cyclic voltammograms of 0.5 mM TP in pH 7.0 PBS at UCPE and MCPE. While no peak is observed at both electrodes in the absence of TP (curves a & b), well resolved oxidative peak with no peak in the reverse scan direction at both the unmodified and modified CPE (curves c & d, respectively) indicated irreversible oxidation of TP at both electrodes which is in agreement with previously reported works [30]. In contrast to the unmodified CPE, an oxidative peak with nearly 50% current enhancement at the MCPE may be ascribed to catalytic property of the surface due to the increased conductivity of the surface as demonstrated by its lower charge transfer resistance in the EIS study. This confirmed successful modification of the carbon paste electrode with a material (EDTA salt) that showed catalytic property towards TP oxidation.

#### 3.2.2. Effect of pH on Ip and Ep of TP at MCPE

**Fig 5:**
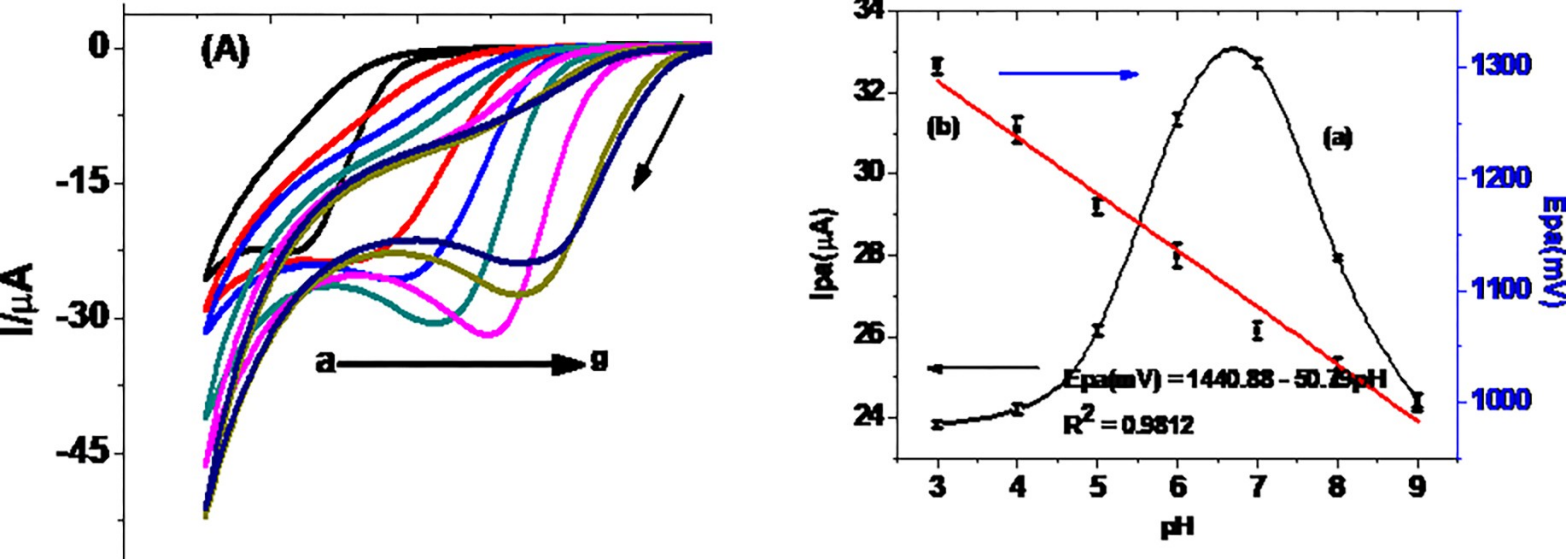
(A) Cyclic voltammograms of MCPE in PBS of various pHs (a-g: 3, 4, 5, 6, 7, 8, and 9, respectively) containing 0.5 mM TP, (B) Plot of average oxidative (a) peak current and (b) peak potential *versus* pH (n 3).

While investigation of the effect of pH on the peak current helps to determine the optimum pH of the buffer (supporting electrolyte) over which maximum current is obtained, its effect on peak potential also helps to survey the proton participation and protons:electrons ratio involved at the rate determining step of the redox reaction. Cyclic voltammograms of 0.5 mM TP in PBS of pHs in the range 3.0–9.0 are presented in Fig 5A. As can be seen from Fig 5B (curve a), the oxidative peak current increased with pH from 3.0 to 7.0 and then decreased afterwards indicating pH 7.0 is the optimum value. The observed trend of oxidative peak current with pH in this study was in agreement with reported trend [30]. The oxidative peak potential of TP also showed shift with pH of the PBS with a slope of 52.5 mV (curve b of Fig 5B), which is very close to the ideal value of 59 mV for participation of protons and electrons in a 1:1 ratio [30, 31].

#### 3.2.3. Effect of scan rate on Ip and Ep of TP at MCPE

**Fig 6:**
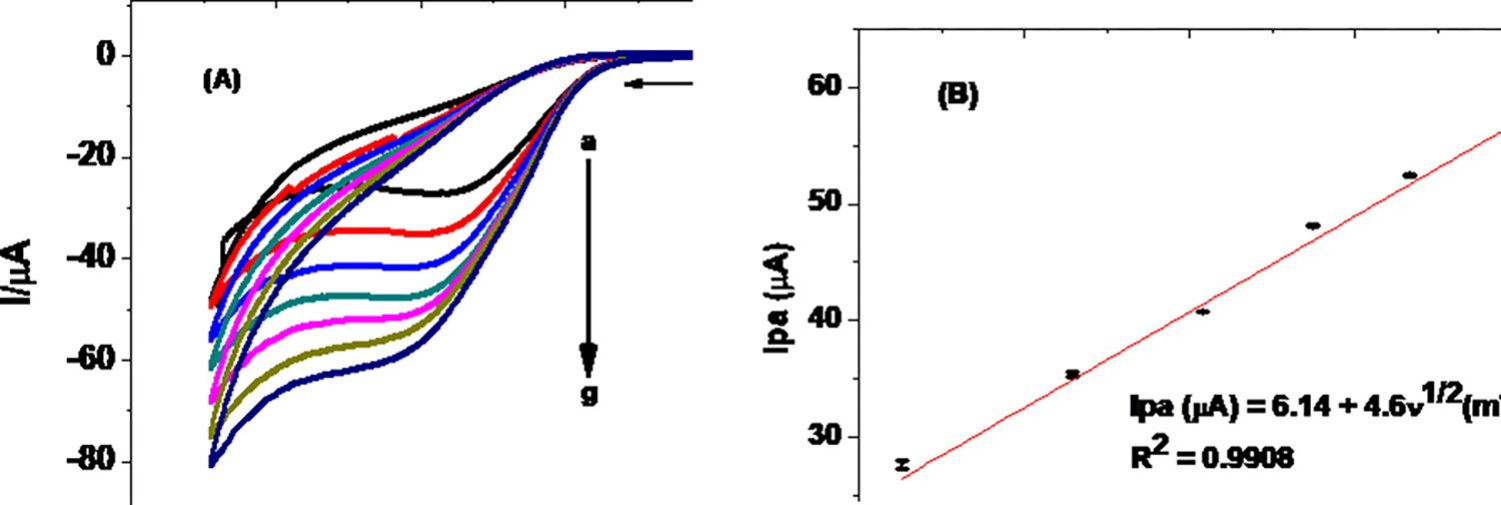
(A) Cyclic voltammograms of MCPE in pH 7.0 PBS containing 0.5 mM TP at various scan rates (a-g: 20, 40, 60, 80, 100, 125, and 150 mV/s, respectively), (B) plot of average Ip vs ʋ^1/2^ (n 3).

Cyclic voltammograms of pH 7.0 PBS containing 0.5 mM TP at MCPE scanned at various scan rates are presented in Fig 6A. While observed potential shift with scan rate is confirmation for the irreversibility of the oxidation reaction of TP at the MCPE, better correlation of the peak current with square root of scan rate (R^2^ 0.9908) (Inset of Fig 6B) than with scan rate (R^2^ 0.9672) (figure not shown) indicated that the oxidation of TP at MCPE was predominantly controlled by diffusion mass transport of TP from the bulk of solution to the electrode/solution interface, which is in agreement with previously reported results [31].

To further confirm that the rate of the MCPE was predominately diffusion controlled, the slope of the regression equation of plot of log (Ipa) against log (ʋ) was investigated.

**Fig 7:**
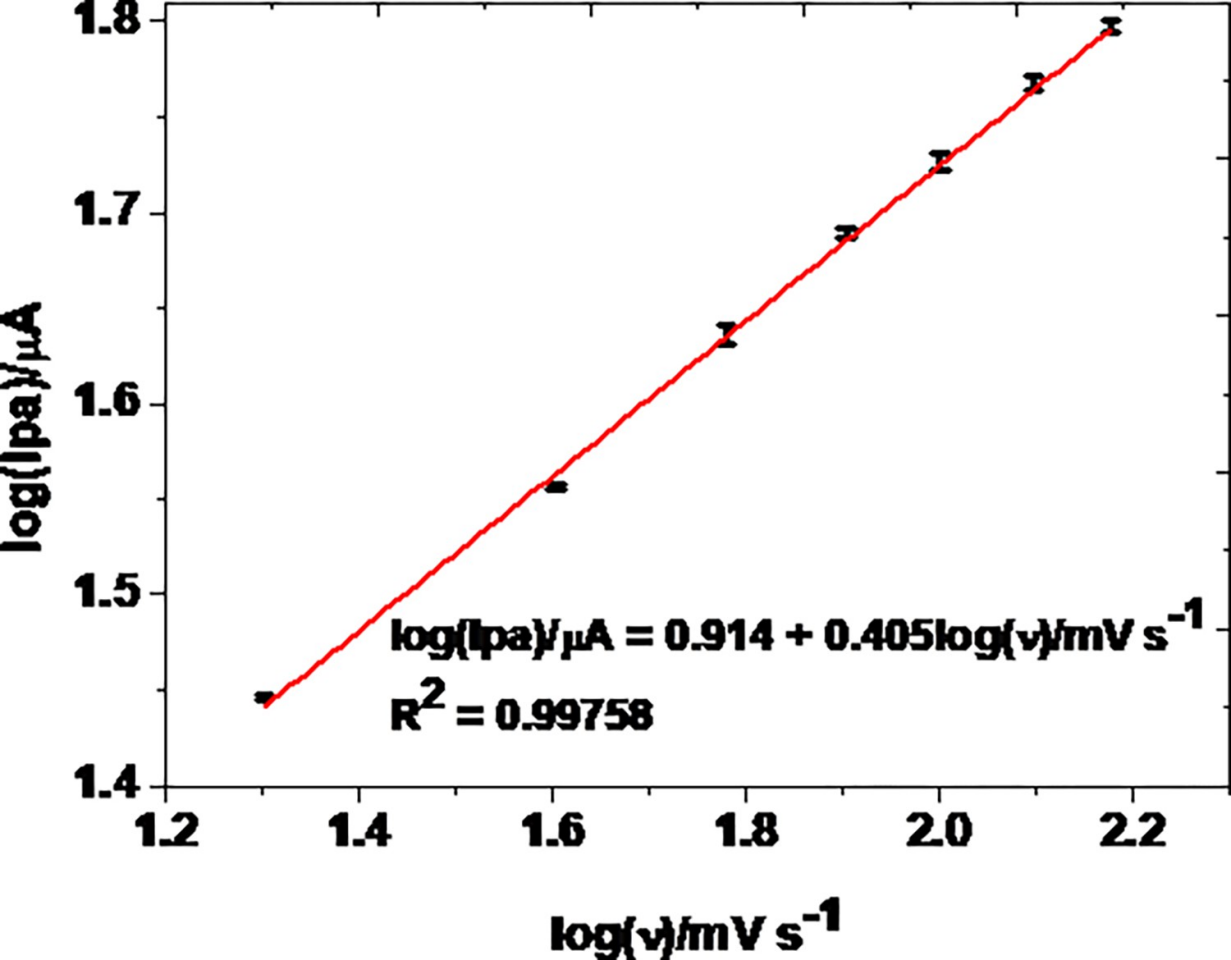
Plot of log of average Ip versus log (ʋ) in the studied range of scan rate (n 3).

**Fig 8:**
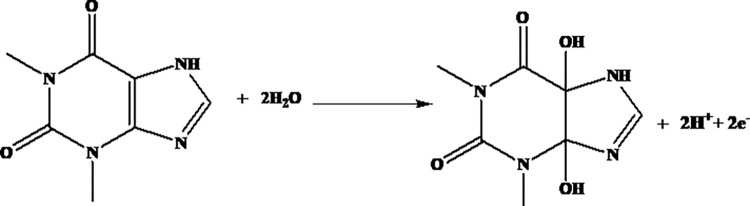
Proposed oxidation reaction mechanism of TP at MCPE.

As can be seen from Fig 7, a slope value of 0.405 for the regression equation of the plot of log(Ipa) against log(ʋ) is close to the theoretical value of 0.5 [34] for a reaction process controlled by diffusion mass transport. The number of electrons involved (n) for an irreversible diffusion controlled reaction is related to the peak potential difference and electron transfer coefficient given by Eq (2) [36]:

$$\Delta E=E_{p}^{}-E_{p/2}$$ (2)

where E_p_ is the potential corresponding to the maximum peak current, E_p/2_ is the potential at half height of the peak current, α is electron transfer coefficient, and n number of electrons participated. Taking curve (e) of Fig 5 (Ep = 1050 mV, and Ep/2 = 994 mV), *αn* was calculated to be 0.85. The α value for an ideal irreversible diffusion-controlled oxidation reaction being 0.5, n was calculated to be 1.7, which is ≈ 2 electrons. Since the proton:electron ratio from the slope of plot of Ep versus pH (curve b of Fig 5B) is 1:1, it was possible for us to propose an irreversible oxidation reaction of TP that involves two protons and two electrons, which is in agreement with previously proposed mechanism (Fig 8) [1, 30].

### 3.3. Square wave voltammetric investigation of TP at MCPE

**Fig 9:**
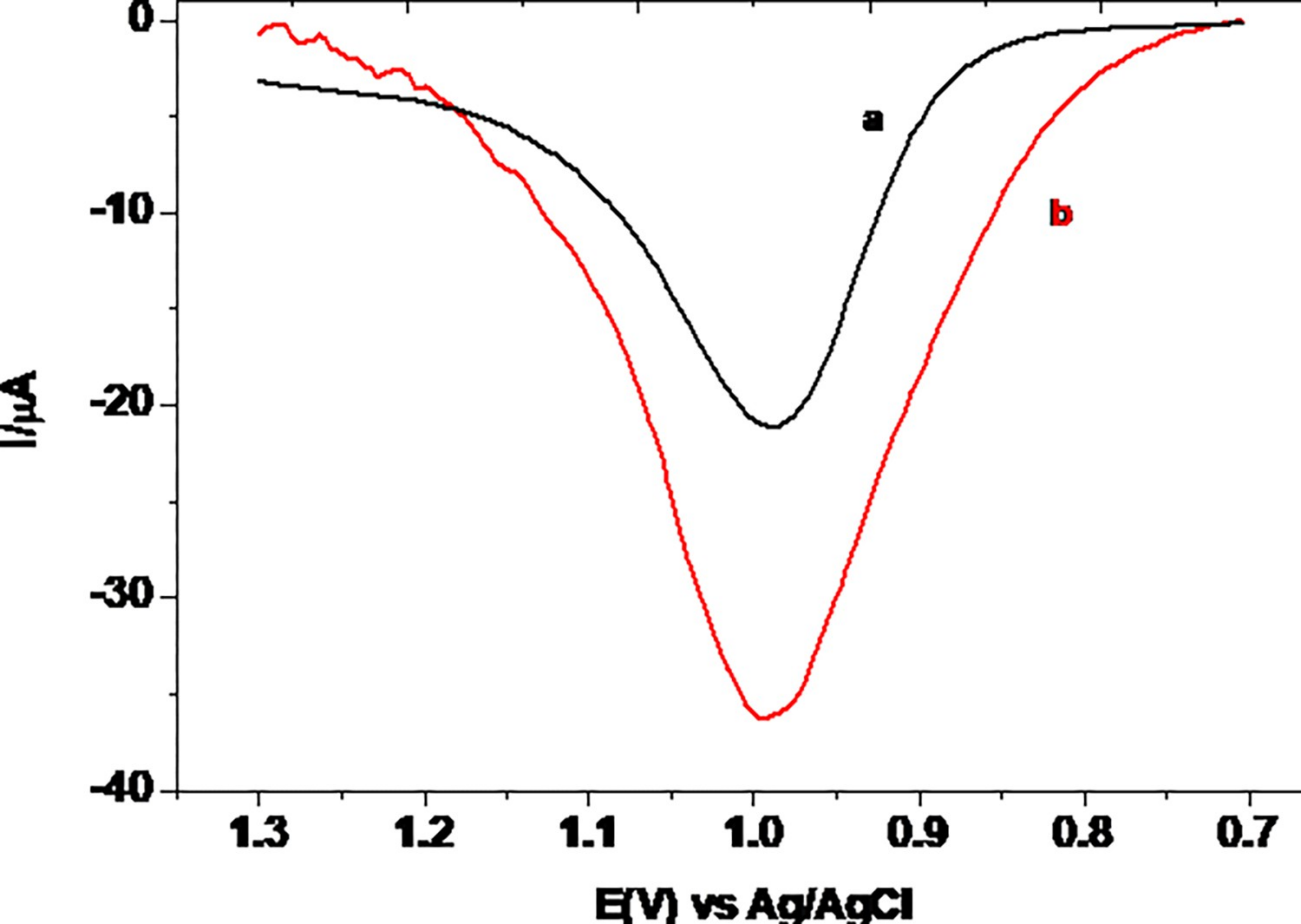
Corrected for blank SWVs of 0.5 mM TP in pH 7.0 PBS at UCPE (a) and MCPE (b).

Square wave voltammetry, which is more powerful to discriminate faradaic from non-faradaic currents than cyclic voltammetry, was used for quantitative determination of TP in pharmaceutical tablet sample. Fig 9 presents square wave voltammograms of 0.5 mM TP in pH 7.0 PBS at UCPE and MCPE. In contrast to the oxidative peak of TP at the UCPE (curve a), appearance of an oxidative peak with over 50% current enhancement at MCPE (curve b) confirmed catalytic property of the modified electrode towards TP oxidation.

#### 3.3.1. Effect of EDTA salt loading

**Fig 10:**
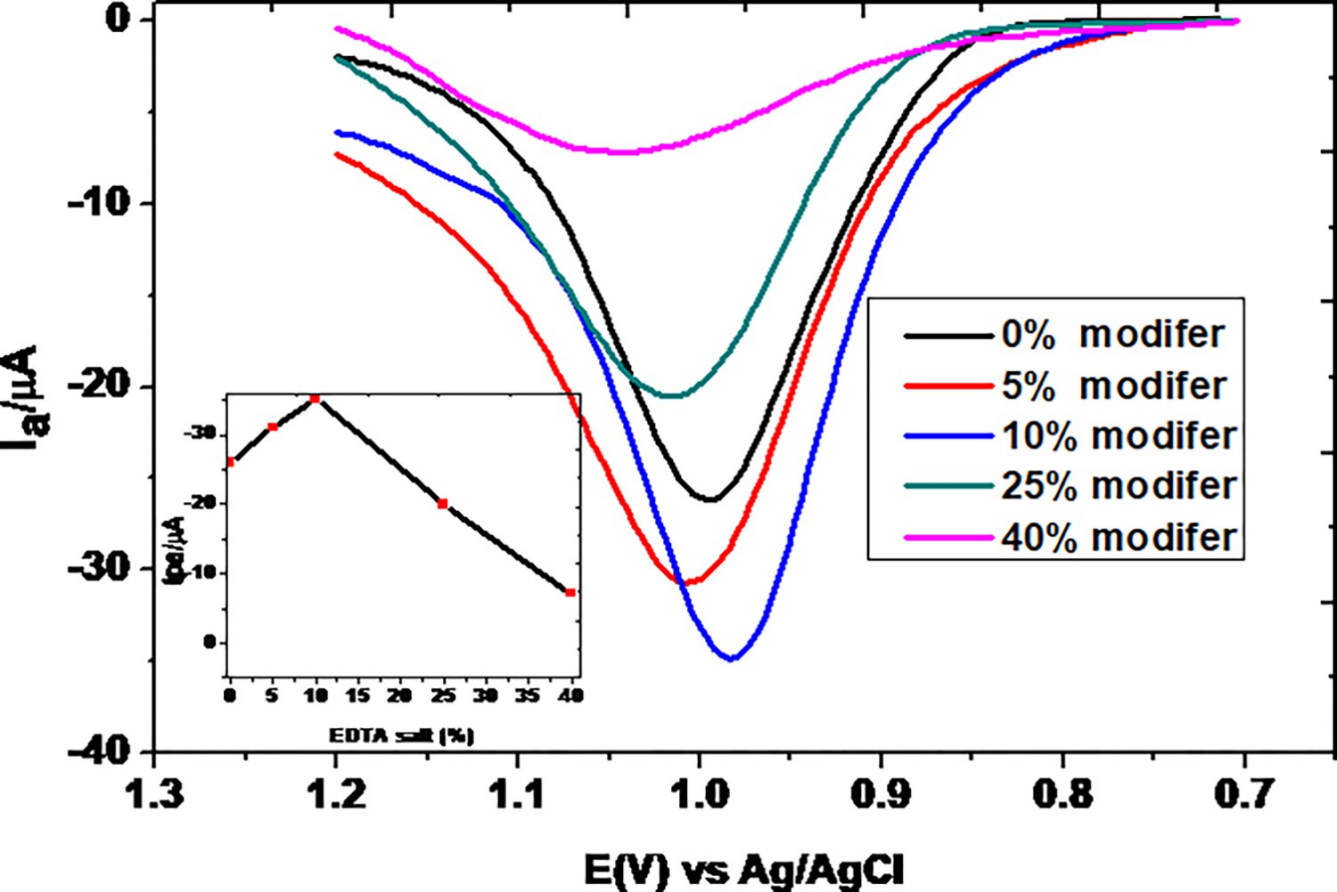
Corrected for blank SWVs of 0.5 mM TP in pH 7.0 PBS at MCPE fabricated with various EDTA salt ratio (0, 5, 10, 25, and 40%).

In order to have the modified electrode that gives the highest current response for TP, the effect of the ratio of the EDTA salt with graphite powder on the current response was studied. Fig 10 presents the SWVs of 0.5 mM TP in pH 7.0 PBS using MCPE fabricated with variable amounts of EDTA salt (0, 5, 10, 25, and 40% of EDTA salt). As can be seen from the inset of the figure, the peak current response increased with the amount of EDTA salt loading up to 10% beyond which it decreased even to the extent below the unmodified electrode. The observed trend might be ascribed to the possible perturbation effect of the modifier on the surface activity of the electrode. In agreement with previously reported work [32], 10% EDTA salt modified carbon paste electrode was taken as the optimum.

### 3.4. Square wave voltammetric calibration curve

**Fig 11:**
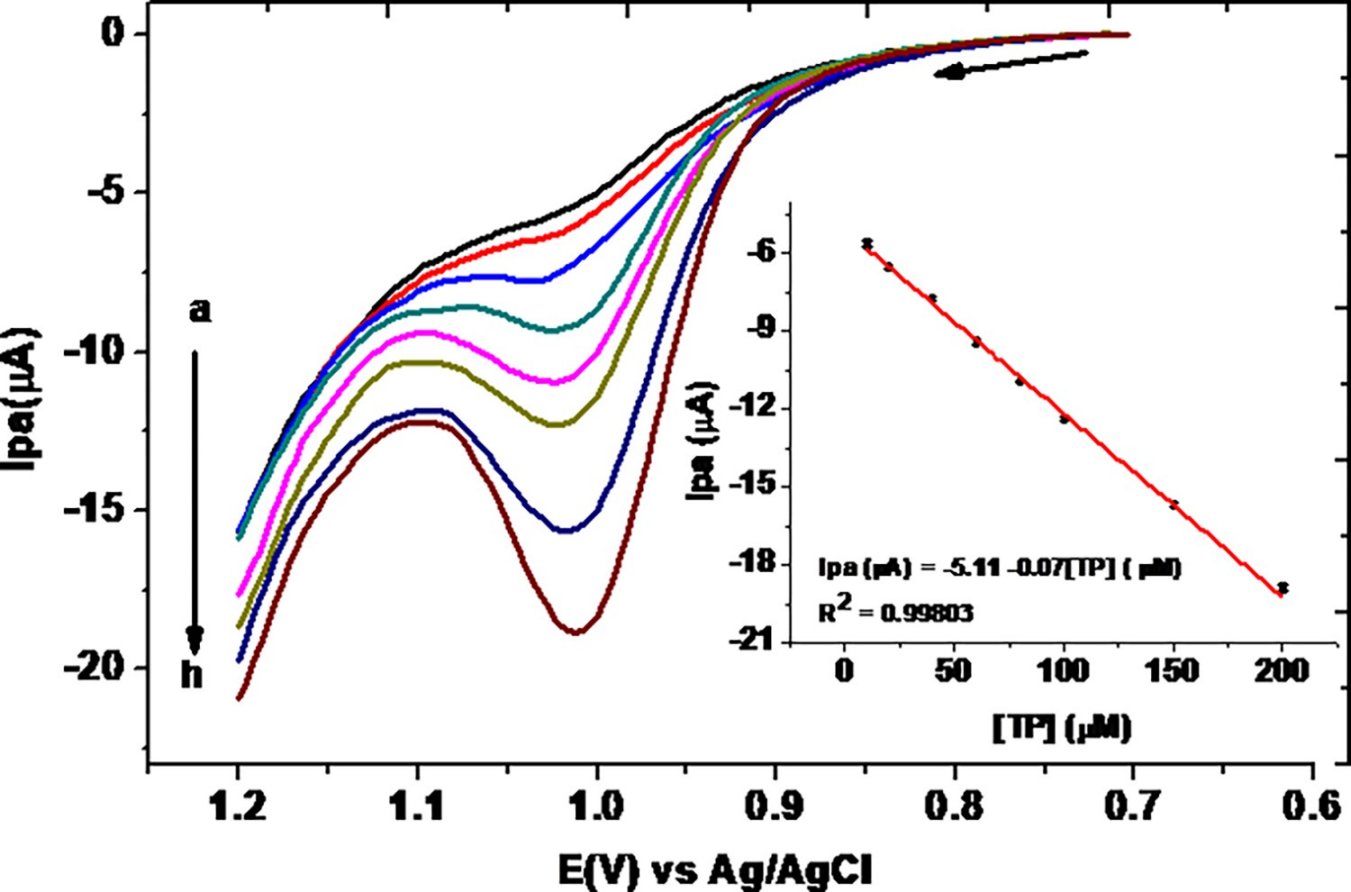
SWVs of 10% MCPE in pH 7.0 PBS containing various concentrations of TP (a-h: 10.0, 20.0, 40.0, 60.0, 80.0, 100.0, 150, and 200 μM, respectively) at scan rate of 100 mV s^-1^ and default SWV parameters (step potential 4 mV, amplitude 25 mV & frequency 15 Hz). Inset: plot of average Ip *vs* concentration of TP (n 3).

Square wave voltammograms of various concentrations of TP in pH 7.0 at 10% MCPE were recorded under the default square wave parameters (Fig 11). The oxidative peak current response of the MCPE showed linear dependence on the concentration of TP in the range 10–200 μM, with a determination coefficient (R^2^), method limit of detection (3S/m), limit of quantification (10S/m) [21] of 0.99782, 2.57 × 10^−2^, and 8.57 × 10^−2^ μM, respectively, where s is the standard deviation of the blank (n 10) and m is the slope of the calibration regression equation.

### 3.5. Determination of TP in tablet sample and recovery test

**Fig 12:**
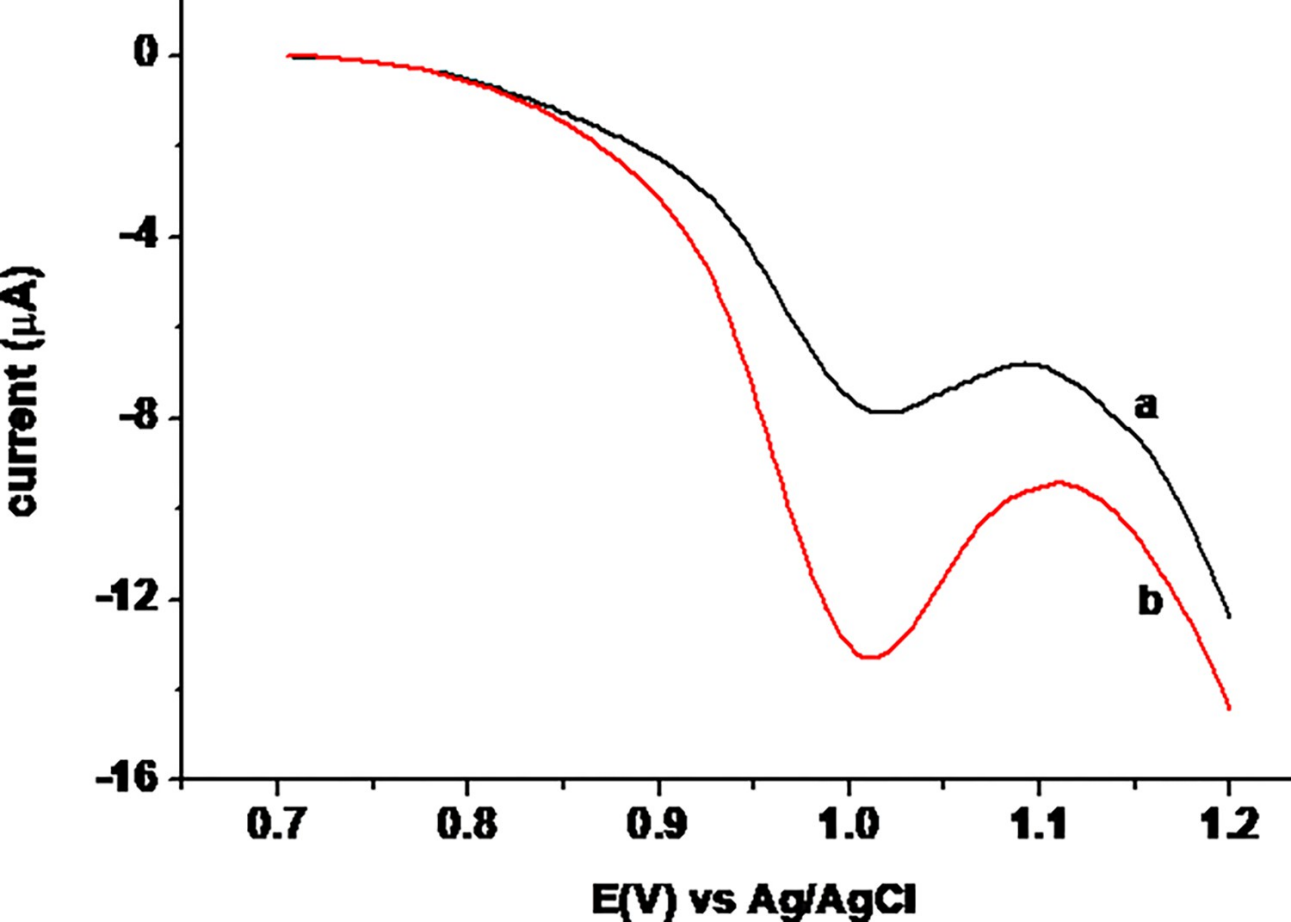
Square wave voltammograms of MCPE in pH 7.0 PBS containing (a) tablet sample with nominal concentration of 39.96 μM, and (b) a + 80 μM standard TP.

The developed square wave voltammetric method was used for determination of TP in Addis pharmaceutical factory (APF) brand TP tablet claimed 120 mg/tablet. The average detected TP level in a tablet sample (curve a of Fig 12), nominally prepared 39.96 μM, was found to be 40.70±1.12 μM (Table 2). Detection of the claimed TP content in the analysed tablet sample with only 2.7% error showed the accuracy of the method and hence reliability of the results.

**Table 2:** Summary of the detected level of TP (mean±SD for n 3) in tablet formulation, spike recovery, and interference recovery results of the developed method.

Sample analyzed	Spiked TP (μM)	Added interferents (μM)			**Detected TP (μM)	Recovery (%)
		AA	Caf	UA		
Tablet sample*	------	------	------	------	40.70±1.12	101.85
Tablet sample*	80	-------	------	------	119±3.52	98.59
Tablet sample*	--	20	--	--	38.05±1.02	95.22
Tablet sample*	--	40	--	--	37.42±1.04	93.64
Tablet sample*	--	--	20	--	40.04±1.63	100.20
Tablet sample*	--	--	40	--	39.94±2.04	99.95
Tablet sample*	--	--	--	20	39.90±2.15	99.85
Tablet sample*	--	--	--	40	39.94±1.95	99.95

*tablet sample claimed 39.96 μM’ AA ascorbic acid; Caf caffeine; UA uric acid

** mean±SD for n 3

### 3.6. Validation of the method

The applicability of the developed method for determination of TP in pharmaceutical tablet formulation was further validated by the spike recovery and interference recovery results.

#### 3.6.1. Spike recovery

Accuracy of the developed method was investigated by analyzing the recovery of spiked 80.00 μM standard TP in the tablet sample claimed to be 39.96 μM (curve b of Fig 12). The method enabled recovery of 98.59% of the spiked 80 μM standard TP (Table 3).

**Table 3:** Summary of comparison of performance of the present method with previously reported methods.

Electrode	Modifier	Method	LDR (μM)	LOD (μM)	Ref.
Screen printed	Graphene quantum dot	DPV	1.0–700.0	0.2	[1]
GCE	ZnO NPs-MWNTs-Cyt	DPV	0.4–15	0.0012	[4]
GCE	MWCNTs	CV	0.3–10	0.005	[5]
GCE	MIP-H-acid	DPV	2–150	0.019	[20]
GCE	P(L-Asp)f-MWCNTs	SWV	0.1–150	0.02	[21]
Gold	MWNTs/poly-L-lysine	SWV	10.0–200.0	2.0	[22]
GCE	RGO-SDS-Nafion	DPV	1–40	0.005	[23]
CPE	CTAB	DPV	0.8–200	0.185	[24]
CPE	EDTA salt	SWV	10–200	0.0257	This work

LDR = linear dynamic range, LOD = method limit of detection, Ref = reference

#### 3.6.2. Interference studies

**Fig 13:**
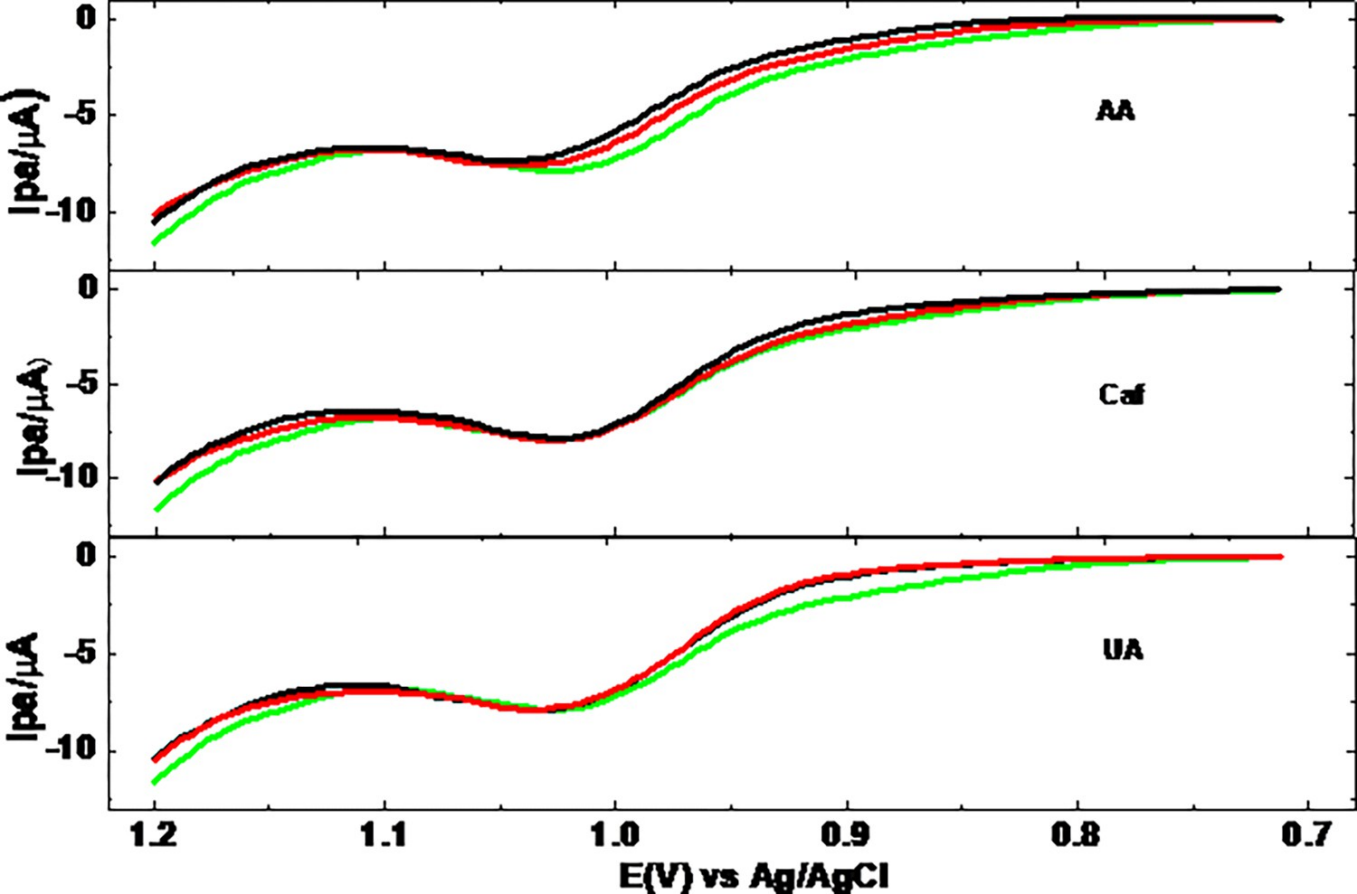
Square wave voltammograms of tablet sample in the presence of 0.0, 20.0, and 40.0 μM of AA, Caf, or UA.

Selectivity of the present method for determination of TP was evaluated by comparing the detected TP in a tablet sample in the absence and presence of selected potential interferents (uric acid UA), ascorbic acid AA), and caffeine Caf). The SWVs of TP tablet sample both in the absence and presence of each interferent at levels of 50, and 100% are presented in Fig 13.

As can be seen from Table 2, the TP level in the tablet sample in the presence of 50, and 100% of the studied potential interferents compared to its level in their absence ranged between 93.64–100.20%. Among the studied potential interferents, while AA at its 100% (40 μM) showed the highest negative error (6.36%), UA at its 50% level showed positive error of 0.20%, which still are in the acceptable range [30].

In general, detection of TP in tablet formulation with an error of 1.85% from the claimed labeled value, spike recovery of 98.59%, and interference recovery result for 50–100% of potential interferents (AA, Caf, and UA) in the range of 93.64–100.20% showed the accuracy, precision, and selectivity of the developed method and hence validated its applicability for determination of TP in a complex matrix sample including tablet formulation.

### 3.7. Comparison of the present method with previously reported methods

The performance of the present method was compared with previously reported methods in terms of the linear dynamic range, detection limit, and of course the type of modifier used (Table 3). In contrast to the previously reported methods which have used moderately expensive substrate and modifiers that demand tedious modification steps, the present study has used the cheapest substrate (CPE) and the most available modifier that requires the simplest modification steps. Moreover, the method showed comparable dynamic range and limit of detection making it a potential candidate.

## 4. Conclusion

CV, EIS and FT-IR results confirmed successful modification of carbon paste electrode with EDTA salt. An irreversible oxidation of TP with an enhanced peak current at the EDTA salt modified carbon paste electrode showed catalytic property of the electrode surface ascribed to the improved surface conductivity as evidenced by EIS result.

Investigation of the effect of scan rate and solution pH on both the peak current and peak potential of TP at the modified electrode revealed a reaction mechanism that involves 2 protons and 2 electrons with diffusion mass transfer controlled kinetics. Detection of 101.85% of the nominal level of theophylline in tablet formulation, 98.59% of spike recovery, and excellent interference recoveries in the range 93.64% (40 μM AA) to 100.20% (20 μM Caf) confirmed the accuracy and hence the reliability of the results obtained using the method. The performance of the present method in terms of the linear dynamic range and limit of detection or sensitivity compared to previously reported works puts the present method an excellent candidate for determination of theophylline in real samples with complex matrix.
